# COVID-19 pandemic—how and why animal production suffers?

**DOI:** 10.1093/af/vfaa059

**Published:** 2021-02-05

**Authors:** Matthias Gauly, Philippe Chemineau, Andrea Rosati, James Sartin

**Affiliations:** 1 Faculty of Science and Technology, Free University of Bolzano, Bolzano, Italy; 2 Department of Animal Science, INRAE, Paris, France; 3 EAAP–European Federation of Animal Science, Roma, Italy; 4 American Society of Animal Science, Birmingham, AL, USA

**Keywords:** coronavirus, livestock, SARS-CoV-2

Nearly a year ago, a novel coronavirus, severe acute respiratory syndrome coronavirus 2 (SARS-CoV-2), named COVID-19, emerged on the world stage. In the ensuing months (to November 2020), the COVID-19 virus has infected 48,947,235 people and resulted in 1,237,417 human deaths (Johns Hopkins University of Medicine Corona Virus Resource Center, 2020). As country by country has succumbed to the pandemic, economic effects have been devastating. Job losses, shortages in production, and lockdowns have resulted in a severe economic challenge for most governments. The International Money Fund (2020) estimated that, by May 2020, government interventions to fight COVID-19 have exceeded $9 trillion dollars (both for fiscal support and loans). One of the critical effects of the pandemic has been a negative impact on agricultural food production and distribution. This issue of *Animal Frontiers* will investigate the problems of pandemics and, specifically, COVID-19 on global animal agriculture.

Global animal pandemics have been a frequent occurrence and have yielded some notable strategy developments, but there is much remaining to be learned and applied. Perhaps the experience gained from the previous pandemics (e.g., the SARS-Pandemic 2002/2003 and the MERS epidemic 2012), as well as the current pandemic can serve as models to assist in the development of approaches to handle future pandemics. Shi et al. (2021) have examined the impacts of various swine disease pandemics and discuss the methods employed in which government, industry, veterinarians, and scientists have worked together to prevent and manage animal pandemics. Furthermore, the appearance of the COVID-19 pandemic in addition to the existing animal pandemics in some countries has further exacerbated the impacts of COVID-19 on animal agriculture.

As the COVID-19 pandemic moved across the planet, there were differing effects of the disease on different countries and industries that, in turn, were often managed in different ways. Pig production in Europe was impacted by two concurrent pandemics, African Swine Fever and COVID-19. The negative effects were associated with decreased demand for pork in Europe and an inability to export products to other countries. The reduced demand for products resulted in an elevated pig population on farms in Europe and elsewhere (Millet et al., 2021). In Australia, the panic buying of meat products by consumers and the COVID-19 infections in processing plant workers slowed processing capacity. In addition, there was a decreased demand for meat products from restaurants and the simultaneous closure of national borders that reduced the export of products. These events created a cumulative effect to increase on-farm animal populations and increased costs to farmers (D’Souza and Dunshea, 2021). In the United States, a similar consequence to the COVID-19 pandemic was observed. The large increase in farm swine numbers presented a challenge to the industry (Tokach et al., 2021). In an effort to avoid mass euthanasia of excess animals, producers, industry, and scientists worked together to develop management and nutritional approaches to delay the entry of swine to processing plants to wait until market conditions recovered.

Along with most other countries, Argentina faced the pandemic by ordering a strict nationwide quarantine and severe restrictions on human contact as a means to prevent the spread of the virus. Argentina has had a little disruption in animal agriculture, in part because of the ability to move beef products from traditional markets to other countries (Arelovich, 2021). However, the economic conditions in Argentina have worsened and this may yet have a consequence for animal agriculture. Similar to many countries, the United States faced an abrupt decrease in the foodservice sector, coupled with overpurchase of goods by concerned consumers and a subsequent disruption in supply chains that were unable to respond quickly to the crisis (Peel, 2021) (Figure 1). The effect of COVID-19 infections in the workforce served to reduce cattle processing leading to more shortages for consumers (Peel, 2021). COVID-19 also impacted economics in China. China implemented travel restrictions, which had serious effects on the normal supply of materials, sales, and transportation and eventually caused disruptions in supply chains in and outside of China (Ding et al., 2021). The prices for livestock and meat rose by 80.8% and pork prices rose by 122.5%. In addition, the global effects of COVID-19 produced severe disruptions to the normal import and export of animal feed and products. Similarly in Ghana, COVID-19 resulted in severe disruptions in importing protein, as well as effects on feeding, management, and disease control (Obese et al., 2021). One consequence was a shortage of feed ingredients for animals. This has all led to an increase in prices for meat and other products in Ghana and a lowered profit margin for farmers (Obese et al., 2021). Although milk and cattle processing were unaffected in the Czech Republic, the closure of farmers’ markets, restaurants, and schools, like in many other European countries, have impacts on foodstuff and cattle prices (Brzakova et al., 2021). Moreover, the quarantine has reduced available farm labor producing additional complications. The result is a need for government supports for farmers and slaughterhouses.

**Figure 1.**
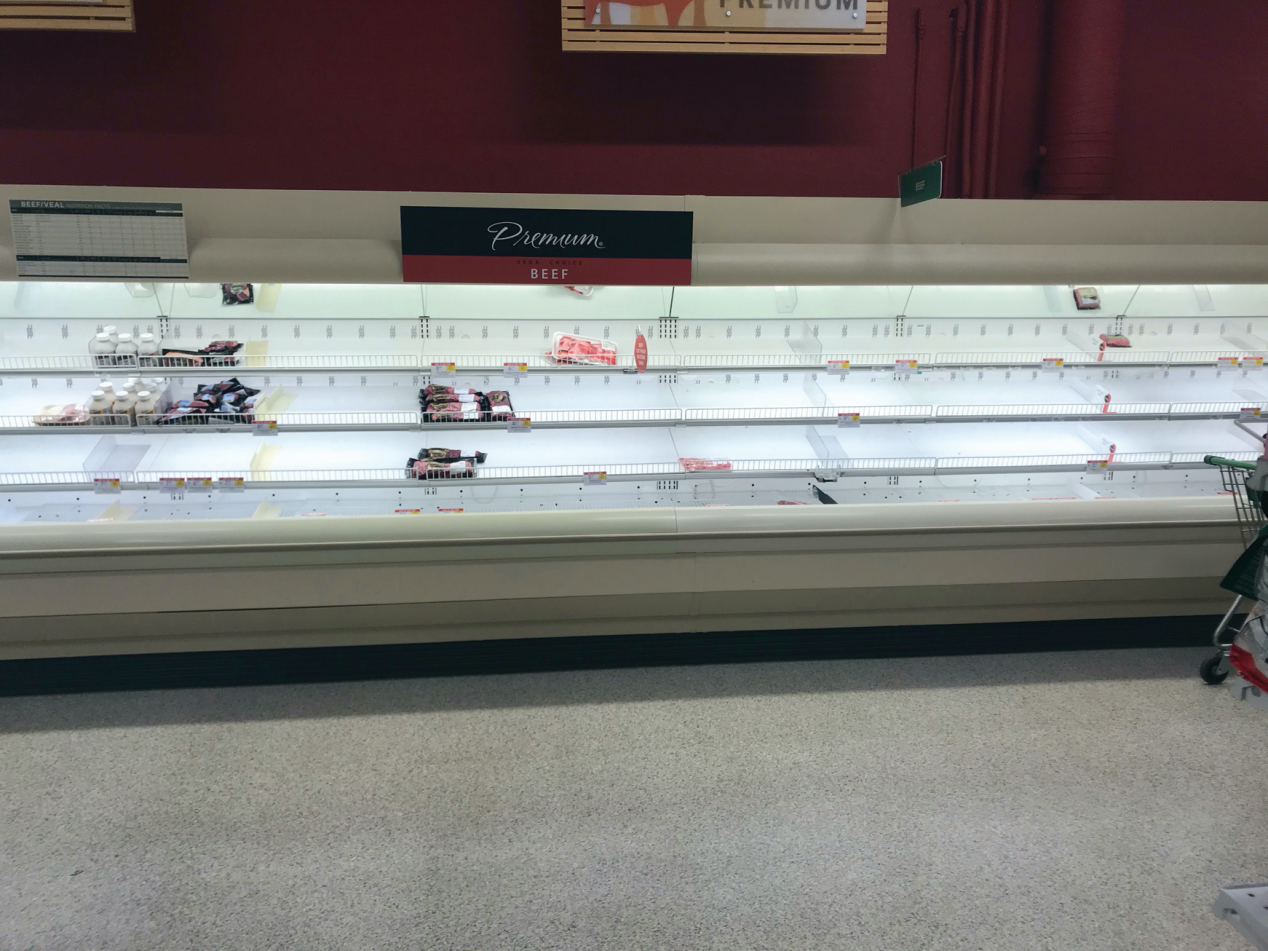
Meat section of a local grocery store showing the lack of meat for sale, March 14, 2020.

Investigation of the effects of the pandemic on specific segments of the animal industry has revealed a number of consequences of the COVID-19 pandemic. The breeding industry faces problems from decreased breeding records and reductions in government supports (David, 2021). In the genetics area, the effects are not yet known (Gandini and Hiemstra, 2021) but, clearly, in both breeding and genetics, there were disruptions in education, mobility, restrictions of movement of goods and supplies across borders, disruptions in international trade, and the need to work from home and away from critical interactions with colleagues (Semianer and Reimer, 2021). Likewise, the pandemic had a little direct effect on camel production, though secondary effects, such as workers becoming infected or shortages of labor across national borders were certainly an issue (Nagy et al., 2021).

Although all countries have experienced significant illness and death of their citizens, market disruptions, business closures, and job losses, not all countries have faced the same consequences to animal agriculture. In addition to the direct impacts on animal production and industries, there were also consequences, such as university closures, reduced research, funding issues, scientific society meetings canceled, etc. The articles in this issue of *Animal Frontiers* both describe the similarities between countries’ responses to COVID-19 and highlight some differences in strategies developed by different countries to deal with the pandemic, particularly in regard to animal agriculture. As this issue is compiled, some countries are emerging from the pandemic, while others are entering a second wave of infections. It is hoped that these articles may provide an accounting of the impacts on animal agriculture, as well as suggest strategies to employ in future epidemics.

## About the Authors

**Matthias Gauly** is an animal scientist and veterinarian. He is a full professor in Animal Science at the Faculty of Science and Technology at the Free University of Bolzano, Italy. He has been active, for over 20 yr in the areas of animal husbandry and management of various livestock species. His current research involves studies on the following: indicator-based evaluation and further development of husbandry systems in terms of animal health, animal welfare, behavior, performance, and economy. He served from 2016 to 2020 as President of the European Association of Animal Science. He has been a Member of the scientific board of the German Federal Ministry of Food, Agriculture and Consumer Protection, since 2009.

**Philippe Chemineau** was initially an Agronomist. He has a PhD in reproductive physiology of domestic animals. He is Emeritus Director of Research INRAE (France) and President World Association of Animal Production. He has been Head of the INRAE Division Animal Physiology and Livestock Systems; member of national INRAE Management Board; Head of the “Delegation for scientific expertise, Foresight and Advanced studies (DEPE)” INRAE Paris; and Head of the INRAE “Direction of Regional policy, Higher education and Europe (DARESE).” He has also been President of European Association of Animal Production, the European Federation of Animal Science. He has published 196 publications and has an h-index: 38.

**Andrea Rosati** is an animal scientist. He obtained the MS and PhD degrees from the University of Nebraska–Lincoln. Since 2002, he has been the Secretary General of the European Federation of Animal Science (EAAP) and World Association for Animal Production (WAAP). He was active before as a geneticist in the Italian Association for Animal Breeders, he managed LGS, the Italian Laboratory for Animal Genetics, and he had also been a member of nine scientific boards of animal breed associations. Since he began to work for EAAP and WAAP, he has been active in animal science dissemination managing the European Annual Meeting of Animal Science, creating three new journals, and other activities, including leading EU-supported projects related to animal science and livestock industry.

**James Sartin** received a PhD in physiology with an emphasis in metabolic endocrinology. He is an Emeritus Professor at Auburn University and serves as the Editor in Chief of *Animal Frontiers*. He is a Past President of the American Society of Animal Science and a Past President of the World Association of Animal Production. He is a founder and first Editor of Domestic Animal Endocrinology and a past Editor of the *Journal Animal Science*, *Animal Frontiers*, and *Translational Animal Science*.
